# Design and implementation of an academic enrichment program to improve performance on the internal medicine in-training exam

**DOI:** 10.1080/10872981.2019.1686950

**Published:** 2019-11-11

**Authors:** Amr Dokmak, Amr Radwan, Meredith Halpin, Bertrand L. Jaber, Claudia Nader

**Affiliations:** a Department of Medicine, St Elizabeth’s Medical Center, Tufts University School of Medicine, Boston, MA, USA; b Division of Hematology & Medical Oncology, Department of Medicine, Boston University School of Medicine, Boston, MA, USA

**Keywords:** Medical education, internal medicine residency, academic enrichment program, in-training exam

## Abstract

The internal medicine In-Training Exam (ITE) is administered at residency training programs to assess medical knowledge. Our internal medicine residency program witnessed a performance decline on the ITE between 2011 and 2014. The goal of this quality improvement project was to improve medical knowledge among residents as measured by an improvement in performance on the ITE, through the design and implementation of an Academic Enrichment Program (AEP). The AEP was designed in 2014–2015, and entailed a multipronged approach, including strengthening and tailoring of the didactic curriculum, establishment of a minimum conference attendance rate, and adoption of the *New England Journal of Medicine Knowledge-Plus* Internal Medicine Board Review platform. Residents performing below a pre-specified percentile rank cutoff on the previous year’s ITE in any of the 12 content areas were required to complete a pre-specified percentage of the question bank in that specific topic. We examined a total of 164 residents enrolled in our program under the categorical training track. The mean (± SEM) ITE percentile for the 12 content areas increased significantly from calendar years 2011–2014 to 2015–2018, reflecting implementation of the AEP (p < 0.001). In brief, compared to the AEP-unexposed graduating classes of residents, the AEP-exposed graduating classes of residents displayed a significant improvement in the mean ITE percentile rank. This quality improvement project was carried out at a single institution. The implementation of a structured academic enrichment program significantly improves performance on the ITE.

## Introduction

Promoting self-directed learning during residency faces multiple challenges, as trainees frequently experience a high burden of administrative tasks, including documentation in electronic medical records [1–5]. These competing priorities and tasks allow limited time to balance clinical activities with educational goals within the 80-hour workweek. Our internal medicine residency program witnessed a poor overall performance on the In-Training Exam (ITE) between 2011 and 2014, which was paralleled by a decline in the American Board of Internal Medicine (ABIM) certification exam pass rate for first-time takers between 2013 and 2015. In a study by Willett et al., the majority of program directors across the US attributed a decline in ABIM certification exam pass rates to residents spending less time independently reading to improve their medical knowledge amongst other reasons [6]. Cognitive fatigue has also been shown to influence the performance of students on standardized tests [7].

With a decline in our 3-year rolling pass rate for first-time takers of the ABIM certification exam to less than 80%, we realized that major changes needed to be urgently implemented to reverse this trend. Our goal was to have 100% of our graduates pass the ABIM certification exam on their first attempt as we hoped that achieving the 100% success rate would lead to the generation of more knowledgeable physicians, which in turn would translate to better patient care.

One of the known predictors of ABIM certification exam's first-time success is performance on the ITE. Several studies have identified specific cutoffs for ITE percentile ranking as predictors of successful first-time ABIM certification exam pass [8–11]. Accordingly, residency programs in various specialties have developed a range of curricular interventions, including frequent faculty meetings, instilling educational conferences, developing individualized study plans, and providing online cases, practice questions, group learning scenarios, and customized web-based teaching tools, to improve the ITE scores as means of achieving a higher first-time board examination pass rate [12–23].

In this quality improvement report, we summarize the design and implementation of an Academic Enrichment Program (AEP), aimed at promoting self-directed learning and improving medical knowledge among our residents as measured by an improvement in performance on the ITE and ABIM certification exam. Through a multipronged approach and a series of innovative approaches, including the implementation of a highly interactive web-based platform that incorporates artificial intelligence, we showcase our experience, with the hope that our approach can be served as a road map for other training programs facing similar challenges.

## Methods

### Setting and brief description of the residency program

St. Elizabeth’s Medical Center is a community hospital located in Boston, Massachusetts. It serves as the tertiary care hospital for Steward Health Care and is a major teaching affiliate of Tufts University School of Medicine. The internal medicine residency program houses a total of 54–55 trainees and recruits annually 22 interns (6 in the preliminary track and 16 in the categorical track), and 2 chief medical residents (at a PGY-4 level). Interns matching in the preliminary track are primarily graduates of US medical schools, whereas the majority of interns matching in the categorical track are international medical graduates. The program adopts a 4 plus 2 scheduling block whereby residents rotate on a 4-week inpatient block alternating with a 2-week outpatient block. While the AEP described below focused on residents enrolled in the categorical track and was informed by their individual performance on the ITE, all the residents were active participants and benefited from this program.

The teaching faculty actively participating in resident education consists of 13 primary care physicians (serving as continuity clinic preceptors), 12 subspecialty education coordinators, 34 additional subspecialists (for teaching in both hospital and clinic settings), 3 dedicated teaching hospitalists, and 2 chief medical residents. All the subspecialty education coordinators meet with the program leadership annually to ensure that the subspecialty curriculum correlates with the blueprints of the ABIM internal medicine certification exam and that the clinical and didactic ACGME requirements are met to optimize the residents’ educational experience.

### Description of the academic enrichment program

In academic year (AY) 2014–2015, we developed and implemented a robust AEP that entailed three main interventions: 1) a bolstering of the format and content of the didactic sessions; 2) a minimum conference attendance requirement for residents; and 3) an individualized study plan for residents based on annual ITE performance. The Program Evaluation Committee monitored annually the progress of this initiative, recommending further refinements for the next AY. The following is a detailed description of this three-pronged approach (Table 1).

**Table 1.** Description of the multi-pronged academic enrichment program

AEP Component	Comment
*Bolstering the Curriculum*	
Use of blueprints of the ABIM certification exam for apportioning of subspecialty didactic lectures	Program leadership tracked allocated subspecialty percentages for the annual ABIM certification exam yearly and apportioned the corresponding percentage for each subspecialty didactic lecture out of the total didactic lectures for each academic year
Quarterly didactic conferences by subspecialty topic	Subspecialty topic themed academic quarters with focus on the topic through Formal didactic sessions *NEJM Knowledge-Plus Internal Medicine Board Review* questions according to quarterly subspecialty topics
Weekly conferences dedicated to each PGY level in a more interactive format	PGY1-specific PICO-based reports PGY2-specific case-based workshops PGY3 MKSAP-based board review sessions
*Establishment and Monitoring of a Minimum Conference Attendance Rate Requirement*	
Minimum conference attendance requirement of 80% for all residents (on ambulatory and elective rotations)	Resident not meeting attendance requirement on a quarterly basis required to meet with academic advisor to develop a plan to improve attendance
Collaboration with nursing department to allow for protected time during noon conferences for residents on ward rotations	Pagers signed out to chief residents to respond to emergent and urgent requests only
Recognition and award system for residents achieving >90% conference attendance rate	Reward of a half personal day where the resident is exempted from clinical duties
*Individualized Study Plan based on IM-ITE Performance*	
Adoption of the *NEJM Knowledge-Plus Internal Medicine Board Review* platform/bank of questions	Adaptive learning platform in web and app format based on strongest available evidence Delivery of an efficient and individualized ABIM-focused extensive bank of questions
Completion of monthly subspecialty *NEJM Knowledge-Plus Internal Medicine Board Review* questions	PGY1, PGY2, and PGY3 to complete 30%, 50%, and 100% based on ITE performance threshold
Monthly medical Jeopardy	Per scheduled subspecialty conducted to review and reinforce key learning points

AEP, academic enrichment program; PGY, post-graduate year; IM-ITE, internal medicine in-training exam; NEJM, New England Journal of Medicine; PICO, Problem/Patient/Population, Intervention, Comparison, and Outcome.

### 1) Bolstering the curriculum

Each AY, we updated the didactic curriculum to correlate with the blueprints of the ABIM certification exam, which provides an outline of the content areas and their approximate percentages for the annual exam; in turn, this allowed us to apportion the subspecialty topics accordingly in terms of didactics and curriculum time dedicated to each subspecialty. We assigned topics for the didactic noon conferences on a quarterly basis. We assisted faculty members in preparing for their presentations by providing them with an outline of highly tested topics and areas where residents underperformed on the prior year’s ITE. We also provided content area-related recommendations on high-value care derived from the Medical Knowledge Self-Assessment Program (MKSAP) and the ABIM Foundation Choosing Wisely Campaign. In addition to our daily traditional morning case-based conferences presented by residents under the supervision of chief medical residents and teaching faculty, and afternoon didactic sessions presented by teaching faculty, we added weekly conferences dedicated to each PGY level in a more interactive format. These sessions were a *PGY1 intern report* focusing on the PICO framework (Problem/Patient/Population, Intervention/Indicator, Comparison, and Outcome), a *PGY2 subspecialty-based small group session*, and a *PGY3 board review session*. In addition, the chief medical residents tracked all topics being presented by residents and reviewed them prior to the presentation to ensure that accurate and high-yield information was conveyed in each lecture.

In order to strengthen the ambulatory-based education, we adopted a new ambulatory curriculum using the PEAC (Physician Education and Assessment Center) modules (https://ilc.peaconline.org/) and ensured protected time on Monday mornings during each of the 2-week ambulatory blocks. During these sessions, several relevant topics were discussed in a flipped classroom approach. This strategy ensured that all residents receive standardized didactic ambulatory experience regardless of their continuity clinic site.

### 2) Establishment and monitoring of a minimum conference attendance rate requirement

Our second intervention was the establishment of a minimum requirement for conference attendance. We required that all residents attend a minimum of 80% of the didactic lectures during their ambulatory block and elective clinical rotations. Inpatient-based rotations had individualized requirements by type of conference and rotation. The program leadership worked with the nursing department to allow for protected time during noon conferences for all residents on ward rotations by having all pagers signed out to the chief residents who would respond to urgent requests during these sessions. Any resident not meeting the attendance requirement on a quarterly basis was required to meet with their academic advisor to discuss and develop a plan to improve attendance. There was also a recognition system for residents who attended >90% of the didactic sessions, namely an extra half personal day reward during an upcoming ambulatory block.

### 3) Individualized study plan based on ITE performance

In addition to optimizing the content and exposure to our didactic curriculum, we created a medical knowledge remediation plan targeting the needs of the individual learners over the course of an AY. In AY 2014–2015, we began utilizing the *Access Medicine* platform (McGraw-Hill Medical). Residents performing at <25th percentile on the 2014 ITE in any given content area were required to complete modules with pre- and post-tests, and score >75% in each content area.

In AY 2015–2016, we obtained approval from the Graduate Medical Education office to acquire a new web-based educational tool for our training program by purchasing an annual subscription to the *NEJM Knowledge-Plus Internal Medicine Board Review* platform (https://knowledgeplus.nejm.org/) for all our residents. This is an adaptive learning platform created by >200 physicians from leading academic institutions based on the strongest available evidence. Accessible in web and app format, this platform delivers an efficient, individualized, and adaptive ABIM-focused extensive question bank of over 5,500 questions covering over 2,700 learning objectives. The adaptive component of this bank of questions identifies knowledge gaps and emphasizes areas for improvement. This is achieved by allowing learners to not only answer a question but rate their confidence in their answer (‘I know it’, ‘Think so’, ‘Unsure’ and ‘No Idea’), and then continues to test topics for each learner based on identified areas of weakness. In addition, it has two timed (2-hour) practice exams and identifies and reinforces areas of weakness for each resident. PGY1s, PGY2s, and PGY3s performing at <35th percentile on the previous year’s ITE in any given content were required to correctly complete 30%, 50%, and 100%, of the corresponding content area, respectively. The subspecialty content areas were tackled as monthly themes, and residents were encouraged to use the *NEJM Knowledge-Plus Internal Medicine Board Review* tool to consolidate knowledge in that particular subspecialty irrespective of their ITE performance. At the end of each month, medical Jeopardy for that subspecialty was conducted to review and reinforce key learning points.

The time spent answering questions, the percentage of correct answers, and other learner-specific data were tracked on a dashboard that was accessed by the program leadership. At the end of each month, the chief residents reviewed the data of the assigned module to ensure that the goal was met. Any residents not meeting the goal were required to submit a plan of action to the chief residents, their academic advisor, and the program director. In AY 2016–2017 and 2017–2018, the ITE performance threshold was raised to <40th percentile with the same total requirement for content areas by PGY category.

### Theory of change

Residency is a busy and stressful part of a doctor’s career. Stressors come in different flavors and include extensive work hours, sleep deprivation, neglect of personal relationships, financial struggle, and most importantly patient care responsibilities [24]. In addition to the aforementioned challenges, individual learners have varying levels of self-discipline, study habits, and test-taking skills. We therefore strived to create an AEP that was individualized to each learner with our didactic curriculum and personalized study plan based on ITE performance to improve medical knowledge and study skills, and help learners find time to study. Our hope was that by incorporating a self-study structure into the academic year, residents would develop organizational and study skills to help improve their medical knowledge as measured by performance on the ITE, gain lifelong learning habits, and better navigate some of the challenges they face during their residency.

### Evaluation plan and analyses

We monitored our intervention by examining the program performance on the ITE over an 8-year period, the latter half of which reflected the implementation of the AEP. We trended our 3-year rolling pass rate for first-time takers of the ABIM certification exam over a 6-year period, the latter half reflecting the implementation of the AEP. We also conducted an annual internal survey of residents spanning AY 2015–2018 on their perception of the *NEJM Knowledge-Plus Internal Medicine Board Review* platform, using 1–5 Likert scale (1 = Poor; 2 = Fair; 3 = Good; 4 = Very Good; 5 = Excellent).

For the analysis, the annual percentile ranking on the 12 content areas of the ITE was averaged and trended over an 8-year period with the last 4 years reflecting our AEP (Table 2).

**Table 2.** Annual percentile ranks for each of the 12 content areas on the in-training exam (ITE)

	Pre-implementation of AEP				Post-implementation of AEP			
ITE content area	2011	2012	2013	2014	2015	2016	2017	2018
Cardiology	10	15	9	26	31	52	84	88
Endocrinology	33	17	18	9	33	49	49	92
Gastroenterology	15	30	12	21	33	48	52	86
General internal medicine	13	6	12	12	35	35	34	76
Geriatrics	7	10	16	5	34	39	12	74
Hematology Oncology	5	25	18	38	40	37	72	82
Infectious Disease	12	9	25	15	59	52	66	75
Nephrology	59	14	19	26	60	56	56	72
Neurology	5	6	6	5	33	36	61	63
Pulmonary and Critical Care	12	19	28	36	41	64	44	94
Rheumatology	12	8	10	16	27	36	57	79
High-Value Care	N/A	7	16	21	36	46	53	88

AEP, academic enrichment program.

Comparisons of continuous variables were performed using the nonparametric Friedman’s two-way analysis of variance test and Mann–Whitney U test. Continuous variables are described as mean ± standard error of the mean (SEM), and categorical variables as count and percentage. All analyses were performed using the SPSS statistical package version 22 (IBM Corporation, Armonk, New York). A two-sided P value of less 0.05 was considered statistically significant.

The institutional review board determined that this quality improvement project (No. QI029) was not considered human subjects research.

## Results

We examined a total of 164 residents enrolled in our program under the categorical training track. At the time of enrollment, the mean age was 29 ± 0.3 years, 46% were women, 82% were graduates of international medical schools, and mean time from graduation to start of residency was 2.7 ± 0.2 years. Ninety-seven (59%) residents were exposed to the AEP. There were no significant differences in the baseline characteristics between unexposed and AEP-exposed residents.

**Figure 1.**
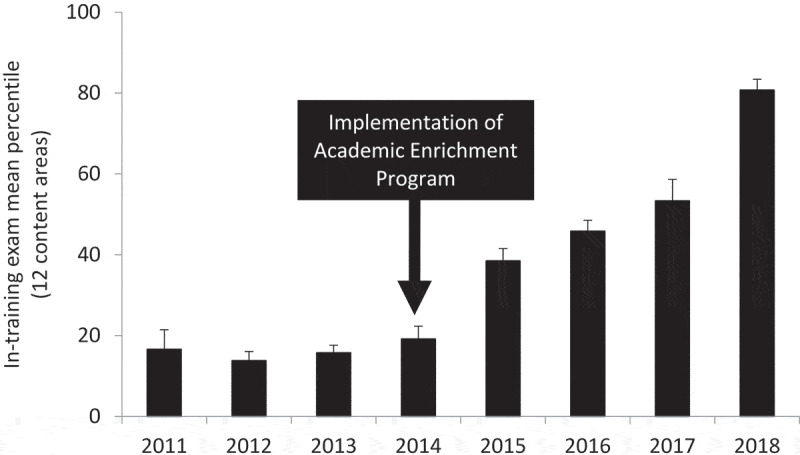
Performance on the internal medicine in-training exam over the course of the implementation of the academic enrichment program. The trended data represent the mean percentile for all 12 content areas over 8 years. P < 0.001 by ANOVA

**Figure 2.**
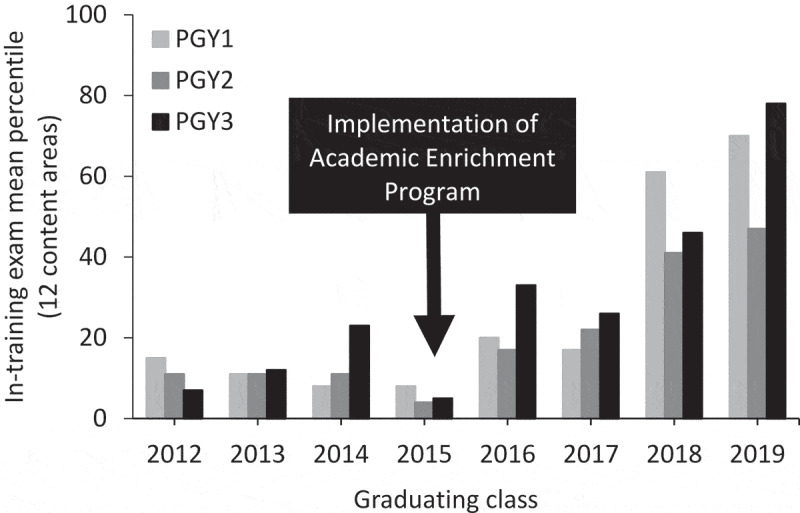
Performance on the internal medicine in-training exam by graduating class and post-graduate year (PGY) level. The trended data represent the mean percentile for all 12 content areas (P = 0.02 by ANOVA for all PGY levels)

**Figure 3.**
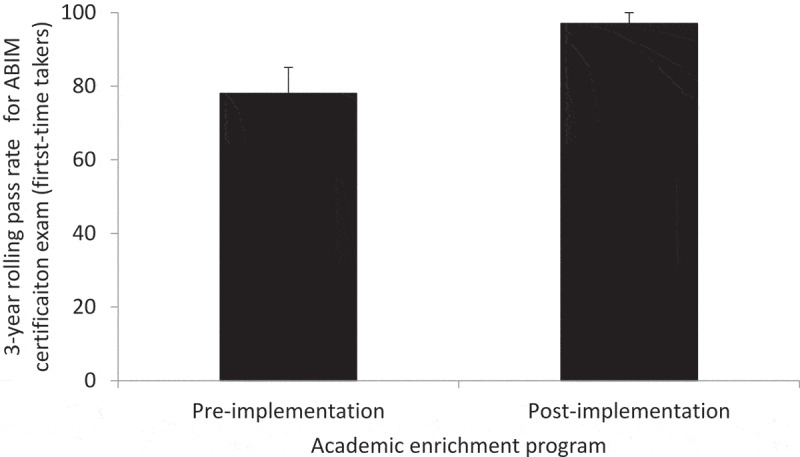
Three-year rolling pass rate for first-time takers of the ABIM internal medicine certification exam pre- and post-implementation of the academic enrichment program. P = 0.12

Table 2 displays the annual percentile ranks for each of the 12 content areas on the ITE over the course of the study period. In brief, following implementation of the AEP, there was sustained improvement in the percentile ranks across all content areas. As shown in Figure 1, following the implementation of AEP, the mean ITE percentile for the 12content areas increased steadily from 2011–2014 (pre-AEP implementation) to 2015–2018 (post-AEP implementation) (P < 0.001). In brief, compared to the AEP-unexposed 2012–2015 graduating classes of residents, the AEP-exposed 2016–2019 graduating classes of residents displayed a significant improvement in their mean ITE percentile rank, including PGY1s (10.5 ± 1.7 vs. 42.0 ± 13.7; P = 0.02), PGY2s (9.3 ± 1.8 vs. 31.8 ± 7.3; P = 0.02), and PGY3s (11.8 ± 4.0 vs. 45.8 ± 11.5; P = 0.02; Figure 2). We next examined the impact of our intervention on the 3-year pass rate on the ABIM certification exam for first-time takers. One hundred (94.3%) eligible residents took the certification exam. Compared to the AEP-unexposed 2012–2015 graduating classes, the AEP-exposed 2015–2018 graduating classes had a higher pass rate on the certification exam; however, this did not reach statistical significance (78%±7% vs. 97%±3%; P = 0.12; Figure 3).

On our annual internal survey, residents rated the *NEJM Knowledge-Plus Internal Medicine Board Review* platform, on a scale of 1–5, as a mean of 3.6, with 84% rating the tool as good-to-excellent.

## Discussion

Our quality improvement project investigates the sustained effect of a multi-faceted curricular approach, the AEP, designed to improve a declining trend in the performance of our internal medicine residency program on the ITE and ABIM certification exam.

As a requirement by the Accreditation Council for Graduate Medical Education (ACGME), the ITE is administered annually to objectively assess resident medical knowledge [25]. Detailed score reports with absolute percent correct answers, percentile rank compared to similar PGY level nationally, and feedback on performance in different content areas are provided to residents thereafter to gauge areas of strength and weakness [26]. While the questions on the ITE are written at the level of proficiency expected of a PGY2 resident, the exam helps determine areas of weakness at any PGY level while there is still time for corrective action. The ABIM certification examination pass rate for first-time takers is considered a quality indicator by the ACGME, with a minimum requirement of 80% pass rate of graduates from the most recently defined 3-year period [25].

As previously mentioned, residency programs in various specialties have developed a range of curricular interventions to improve the ITE scores as means of achieving a higher first-time board examination pass rate [12–23]. Drake et al. have reported on a web-based directed reading program with an aim to increase medical knowledge and reduce ABIM exam failure rate [23]. Their strategy involved identifying incorrectly answered ITE educational objectives and encouraging residents to type a ‘reflection’ representing their understanding of the educational objective. The reflection was reviewed by a faculty preceptor who rated the response as satisfactory or unsatisfactory. The approach adopted by these authors involved one dimension, namely, the web-based platform. Their approach also relied on the subjective opinion of the assigned faculty preceptor. In another study, McDonald et al. demonstrated that improved conference attendance leads to improvement in ITE score [13].

Our AEP allowed trainees to enhance their medical knowledge through different platforms that guaranteed exposure to high-yield internal medicine topics. This was entirely performed under the direct supervision of faculty, who were provided with tools to devise an action plan such as the *NEJM Knowledge-Plus Internal Medicine Board Review* and ABIM certification exam blueprints. Our experience resulted in a significant improvement in ITE scores in the group of residents exposed to the AEP as compared to those who were not. This was observed across all 12 subspecialty content areas described in the ITE and for each individual PGY class. Given that previous studies have demonstrated a direct correlation between performance on the ITE and ABIM certification exam pass rate for first-time takers in internal medicine [8–11,27], we hypothesized that the AEP, among other factors, would have a downstream favorable impact on ABIM certification amongst exposed residents, as shown by a non-significant trend towards a higher 3-year rolling pass rate for first-time takers. Similar correlations have been observed in other specialties, including emergency medicine [28], surgical specialties [29–32], and obstetrics and gynecology [33].

The implementation of our robust AEP required buy-in from all stakeholders and teamwork. In terms of time commitment for residents, there were two daily 1-hour didactic sessions. Conference attendance was required for residents on ambulatory and elective rotations given that the clinical demands on these rotations would usually allow for such educational activities. Residents in need of completing a pre-specified percentage of questions from the *NEJM Knowledge Plus Internal Medicine Board Review* platform based on their most recent ITE score were given 4–5 weeks to complete the task. On average, a task required approximately 1 hour per week per resident. The chief medical residents dedicated 1–2 hours per week to ensure compliance with the measures being monitored (conference attendance rate and the progress and completion of individualized study plan) and to meet with residents who required further assistance. In terms of time commitment for faculty, program leadership meetings were held for 1 hour on a bi-weekly basis. In addition, members of the Clinical Competency Committee, who serve as academic advisors, met regularly with residents and played an important role in reinforcing adherence to the AEP, including conference attendance and timely completion of the assigned individualized study plan. The only incremental cost incurred for developing and executing the AEP was solely for subscribing annually each of our residents to the *NEJM Knowledge Plus Internal Medicine Board Review* platform. The current listed annual subscription to the *NEJM Knowledge Plus Internal Medicine Board Review* platform for residency programs can be found on their website (https://knowledgeplus.nejm.org/products/internal-medicine-board-review-pricing/). Overall, we believe that the aforementioned time investment by all stakeholders and the nominal financial commitment for the successful deployment of the AEP was not too onerous and very reasonable. Furthermore, our multipronged approach, specifically the adoption of the *NEJM Knowledge Plus Internal Medicine Board Review* web-based platform, provided a sophisticated and structured delivery of medical knowledge based on artificial intelligence algorithms that can be easily monitored by the chief residents, making the intervention applicable to other residency programs with minimal time commitment from faculty.

Strengths of our quality improvement project include a large number of residents and detailed data gathered over the span of 8 years. To our knowledge, the multipronged approach that we adopted, including the use of a highly interactive web-based platform incorporating artificial intelligence, had not been previously attempted. Our AEP approach was tailored for each resident based on previous ITE performance, which ensured a more personalized educational plan. In addition, the adoption of the *NEJM Knowledge-Plus Internal Medicine Board Review* platform enhanced didactic curriculum, and establishment of a minimum conference attendance rate provided a wider net and ensured that each resident found a modality that worked best. In other words, our diversified AEP portfolio entailing a multipronged platform provided a higher likelihood of success since different residents have variable learning styles.

Our quality improvement project has important limitations. It was carried out at a single institution, limiting generalizability of the intervention. The results were likely confounded by other resources used by residents for self-study and by the adoption of an enhanced intern recruitment strategy during the intervention period, whereby PGY-1s enrolled in the program during the launch of the AEP, achieved higher scores on the ITE compared to their counterparts who enrolled in prior years. Our resident survey, unfortunately, did not include reasons behind the rating, such as the half personal day reward. The question inquired about the general experience with the *NEJM Knowledge Plus Internal Medicine Board Review* platform. The reward question will be incorporated in future resident surveys. In addition, while our overarching goal was to instill lifelong learning habits for our residents, our study only focused on improving performance on the ITE and ABIM internal medicine certification exam. However, it is our hope that the critical thinking and reading habits that were instilled and reinforced over the course of 3 years will serve our residents well long term as they become and remain life-long learners while pursuing additional post-graduate training or entering clinical practice.

## Conclusion

The implementation of an individualized, structured, and tailored self-study plan for medical residents in the form of an AEP significantly improves performance on the ITE and contributes to a higher ABIM certification exam pass rate for first-time takers. It is our hope that this approach can help inform training programs facing similar challenges and provide a platform to develop creative solutions.
